# Adult ADHD diagnostic pathways in the United Kingdom: a structured review and policy case for a national commissioning and quality framework for England

**DOI:** 10.3389/fpsyt.2026.1860550

**Published:** 2026-07-21

**Authors:** Marios Adamou, Sarah L. Jones

**Affiliations:** 1 School of Human and Health Sciences, University of Huddersfield, Huddersfield, United Kingdom; 2 South West Yorkshire Partnership NHS Foundation Teaching Trust, Wakefield, United Kingdom

**Keywords:** adult ADHD, commissioning, diagnostic pathways, England, NHS, quality standards, stratified care, unwarranted variation

## Abstract

**Background:**

Adult attention-deficit hyperactivity disorder (ADHD) diagnostic pathways in England are under sustained pressure. Demand has risen, waiting times have lengthened, service models remain fragmented, workforce capacity is uneven, and access and quality vary markedly between localities. Although the core clinical principles of ADHD diagnosis are relatively well established, the organisational conditions needed to deliver them consistently remain less secure.

**Aim:**

To synthesise UK-wide evidence on adult ADHD diagnostic pathways and to develop an evidence-informed policy case for a national commissioning and quality framework for England.

**Design and setting:**

PRISMA-informed structured policy-oriented review of UK-wide evidence on adult ADHD diagnostic pathways, interpreted through a health services and commissioning lens. The review is not a registered systematic review and did not undertake formal risk-of-bias scoring; appraisal was structured and narrative.

**Method:**

A structured literature search was conducted across PubMed/MEDLINE, Scopus, Web of Science, ScienceDirect, SpringerLink, Google Scholar, SciSpace, and ArXiv. Searches were conducted between 2023 and March 2026, focusing on publications from 2010 onwards. Screening and reporting were informed by PRISMA 2020 guidance, adapted for a structured literature review rather than a full systematic review. Eligible sources included peer-reviewed empirical studies, clinical audits, service assessments, mixed-methods studies, qualitative studies, consensus statements, systematic or scoping reviews, and policy documents relevant to adult ADHD diagnostic pathways in UK healthcare settings. Findings were synthesised thematically into pathways and cross-cutting system factors.

**Results:**

Twenty-six sources met the eligibility criteria. The evidence showed marked variation in access routes, referral quality, screening and triage practice, assessment delivery, service configuration, transition arrangements, post-diagnostic interfaces, and supporting infrastructure. Clinical standards for diagnosis were comparatively stable across NICE, the Royal College of Psychiatrists, the UK Adult ADHD Network, and the British Association for Psychopharmacology guidance, yet operational delivery was inconsistent.

**Policy implication:**

England requires a national adult ADHD commissioning and quality framework that defines minimum diagnostic standards, core pathway functions, stratified allocation of expertise, mixed-provider governance, and whole-pathway outcome monitoring. The framework proposed here is conceptual rather than operational: it defines minimum functions, standards, and governance expectations, but it does not yet specify operational criteria for stratification, complexity thresholds, audit mechanisms, or rules for local implementation, which remain matters for the implementation phase.

## Introduction

Attention-deficit hyperactivity disorder (ADHD) is a neurodevelopmental condition associated with persistent difficulties in attention regulation, impulsivity, and activity level (1), with consequences that may extend across education, employment, relationships, mental health, and everyday functioning throughout the lifespan (2). Although ADHD was historically framed mainly as a childhood disorder, it commonly persists into adulthood (3). In England, recognition of adult ADHD has increased substantially over the past decade, driven by greater public awareness, improved understanding of lifespan persistence, refinements in diagnostic frameworks, and the growing numbers of young people moving out of child services (4–8).

This growth in recognition has brought longstanding weaknesses in service provision into sharper view (9). Adult ADHD pathways in England have developed unevenly and often reflect local commissioning history rather than coherent national design. Some areas offer specialist adult ADHD clinics, and elsewhere, pathways depend on community mental health teams, broader neurodevelopmental services, mixed-provider arrangements, or combinations of NHS and independent-sector provision. Referral thresholds, access routes, waiting times, diagnostic processes, and post-diagnostic arrangements vary considerably between areas and, at times, within the same integrated care board footprint (4). As a result, access and pathway quality may be shaped as much by geography and local service configuration as by clinical need.

The policy issue is therefore broader than just waiting times. Delays matter clinically and psychologically, but they are only one visible expression of a wider pathway problem. Across the existing literature, the core difficulties include rising demand, uneven service configuration, inconsistent pathway functions, limited specialist capacity, weak transition arrangements, and variable post-diagnostic provision. These features point to systemic misalignment between need, demand, supply, and commissioned pathway design. In that context, long waits, large lists, and recourse to alternative routes of care become predictable consequences rather than isolated failures (4).

A related concern is that much of the observed variation appears difficult to justify based on patient need, population differences, or legitimate local adaptation. This raises the issue of unwarranted variation. In a national health system, some variation is inevitable and desirable because populations, geographies, and organisational capabilities differ. However, when differences in access, delay, pathway quality, continuity, or outcome appear unrelated to those factors, they become matters of equity, consistency, and public accountability.

NHS England’s all-age autism national framework for assessment services (10) provides a relevant policy precedent. It was developed in response to demand-capacity mismatch, local variation, inconsistent pathway quality, and uncertainty about what integrated care boards should commission. Such framework-based responses are already recognised as legitimate when neurodevelopmental pathway heterogeneity and service pressure reach sufficient scale, and this paper can serve as a foundation for developing similar guidance for adult ADHD.

The focus of this paper on adult services is deliberate. We are aware that children’s services face comparable and, in some parts of the United Kingdom, more acute pressures. For example, in Scotland, some neurodevelopmental pathways have effectively been suspended. The decision to address adults separately reflects the substantive differences between the approaches to child and adult ADHD diagnosis rather than any assumption that children’s services are less affected. For example, in children the developmental history is contemporaneous, school observation and educational records are ordinarily available, parental involvement is routine, and provision is organised around child and adolescent mental health and paediatric pathways with their own statutory and educational links. In adults, the developmental history must be reconstructed retrospectively, corroborative informants may be unavailable, and the pathway apart from the new assessment must have regard to transition into adult services. A single all-age framework would risk obscuring these differences, so a dedicated adult framework is therefore needed, and a corresponding framework for children’s services, while equally necessary, falls outside the scope of this paper.

This paper adds to the literature by making an argument not made by any existing strand of evidence alone. Service mapping studies describe the landscape of provision (11), consensus statements describe the scale and seriousness of system failure (9, 12), and quality frameworks and professional guidance define what a good adult ADHD assessment should involve (13–16). However, none of this literature, taken alone, sets out how an adult ADHD pathway should be commissioned and delivered as a whole. This paper brings those strands together and argues that, read together, they support a commissioning-level conclusion: England now requires a national adult ADHD commissioning and quality framework.

The contribution of the paper is therefore beyond restating that services are under pressure, but to show that descriptive service mapping, problem-focused consensus work, and assessment-quality frameworks converge on a broader policy approach. Once synthesised, they support a model of national action built around minimum pathway functions minimum assessment standards, stratified care, mixed-provider governance, and active management of unwarranted variation.

## Methods

### Study design

This study was a PRISMA-informed structured literature review of evidence on adult ADHD diagnostic pathways in UK services. Screening and reporting were informed by PRISMA 2020 guidance (17), adapted for a structured literature review rather than a full systematic review. A structured review design was selected because the literature is diverse in design, purpose, and evidential form. It includes peer-reviewed empirical studies, clinical audits, service assessments, mixed-methods studies, qualitative research, consensus statements, expert position papers, professional guidance, and policy documents. The aim was not to estimate the pooled effect of a single intervention. Rather, it was to identify the pathway components, service conditions, and organisational factors associated with high-quality and timely adult ADHD assessment, and to consider their implications for service design and commissioning.

### Search strategy

A structured literature search was conducted across PubMed/MEDLINE, Scopus, Web of Science, ScienceDirect, SpringerLink, Google Scholar, SciSpace, and ArXiv. Searches were carried out in March 2026 and were restricted to publications from 2010 onwards, to reflect contemporary practice and service delivery.

Search terms combined descriptors relating to population, condition, pathway, setting, and outcomes. Population terms included “adult” and “adults”. Condition terms included “ADHD”, “attention deficit hyperactivity disorder”, and “attention-deficit/hyperactivity disorder”. Pathway terms included “diagnostic pathway”, “assessment”, “referral”, “diagnosis”, “diagnostic decision”, “service”, “screening”, “triage”, and “care pathway”. Setting terms included “UK”, “United Kingdom”, “NHS”, “England”, “Scotland”, “Wales”, “Northern Ireland”, and “British”. Outcome terms included “quality”, “timeliness”, “waiting time”, “service delivery”, and “standards”. The reference lists of included sources and the outputs of relevant professional and policy bodies were hand-searched to identify additional eligible material.

### Eligibility criteria

Sources were eligible for inclusion if they addressed adults aged 18 years or over undergoing ADHD assessment, or if they reported service-level evidence directly relevant to adult diagnostic pathways. Included sources had to relate to UK healthcare settings, including NHS trusts, specialist clinics, community mental health teams, primary care services, neurodevelopmental services, or UK-based independent providers, where relevant to NHS-funded pathways.

Sources were included if they described, assessed, or reported on diagnostic pathways; identification, referral, and triage processes; pathway structures; timeliness metrics; quality indicators; service delivery models; diagnostic practices; transition arrangements; prescribing support; pre-assessment support; post-diagnostic care; or patient experience from referral to diagnostic decision. Eligible material included peer-reviewed empirical studies, clinical audits, service assessments, mixed-methods studies, qualitative studies, consensus statements, systematic or scoping reviews, and policy or guideline documents with direct pathway relevance. Exclusion criteria were child-only studies without separable adult data, non-UK studies without direct applicability to UK service design, treatment-only studies without pathway relevance, case reports, editorials without substantive service analysis, and sources from unrelated clinical domains.

### Selection and synthesis

Titles and abstracts were screened against the eligibility criteria by two reviewers. Full-text assessment was undertaken independently by both reviewers, with disagreements resolved by discussion against the eligibility criteria. Duplicate records were removed before screening. The main databases used were PubMed/MEDLINE, Scopus, Web of Science, ScienceDirect, and SpringerLink. Google Scholar, SciSpace, and ArXiv were used solely as supplementary sources, recognising that these platforms have known reproducibility limitations compared with indexed bibliographic databases. Supplementary searches were used to identify grey literature, conference outputs, and policy documents that would not have been retrieved from indexed databases alone, and the records identified through these sources are included in the records count reported in the Results. Given the diversity of the evidence base, the included sources were assessed for both methodological quality and relevance to the review question. Clinical audits and service assessments were used to identify concrete pathway features and performance issues. Qualitative studies were used to illuminate service-user and clinician perspectives. Consensus and policy documents were used to identify prevailing standards and assumptions about service provision.

No formal risk-of-bias scoring was undertaken because the included evidence was heterogeneous and included service assessments, audits, consensus statements, professional guidance, and policy documents. Instead, evidential weight was assessed narratively based on design, relevance, and directness. Peer-reviewed empirical studies and national surveys were treated as stronger evidence for service patterns. Single-site audits, service assessments, and conference-level reports were treated more cautiously and used mainly to illustrate local pathway mechanisms or implementation risks.

Data extraction focused on study characteristics, service setting, pathway components, timeliness measures, quality indicators, service delivery models, barriers, facilitators, and reported recommendations. Evidence was synthesised thematically. Findings were organised first by pathway stage, including identification, referral, screening and triage, pre-assessment support, assessment, diagnostic decision, and post-diagnostic interface. They were then considered through cross-cutting system factors, including workforce and training, quality assurance, patient experience, transition, service configuration, commissioning, prescribing support, data infrastructure, and equity. The synthesis was interpretive and explanatory, aiming to identify the conditions under which pathway features appeared to support or hinder timely, high-quality adult ADHD assessment.

The included evidence comprised several distinct categories, namely national service-mapping and survey evidence, drawing on the work of Price and colleagues (11, 19) and the Department of Health and Social Care interim report (4); local audit and quality-improvement evidence; patient-experience evidence; consensus statements and expert position papers; clinical guidelines and quality standards; and policy documents. These categories were treated as carrying different forms of evidential weight. National mapping and surveys were used to describe the landscape of provision; local audits to identify operational gaps between standards and delivery; consensus statements to characterise the scale and seriousness of the problem; clinical guidelines and standards to specify the expected content of an assessment; and policy documents to identify available commissioning levers.

Because the review was intended to inform a policy argument about service design, the final stage of analysis considered the implications of the evidence for national commissioning, minimum diagnostic standards, stratified care, management of unwarranted variation, and infrastructure required to support improvement.

### Definitions

Several terms recur throughout this paper and are used in operational rather than rhetorical senses. A commissioning framework is a national or regional specification of what services are expected to deliver, by whom, to what standard, and under what governance, used by commissioners to define and contract pathways. A quality framework is a complementary specification of the minimum standards a pathway is expected to meet, against which performance can be audited. Minimum diagnostic standards are the minimum content requirements for a defensible adult ADHD assessment. Stratified care refers to the allocation of assessment time and expertise to the clinical complexity of the case. Unwarranted variation refers to differences in access, timeliness, pathway quality, continuity, or outcome that cannot be explained by patient need, population difference, or legitimate local adaptation. Whole-pathway commissioning refers to the commissioning of all pathway functions from identification through post-diagnostic support, rather than commissioning of the assessment alone. Mixed-provider governance refers to the governance arrangements that ensure quality, reporting, prescribing, handover, and follow-up are aligned across NHS and independent-sector routes.

## Results

### Search yield and study selection

**Figure 1:**
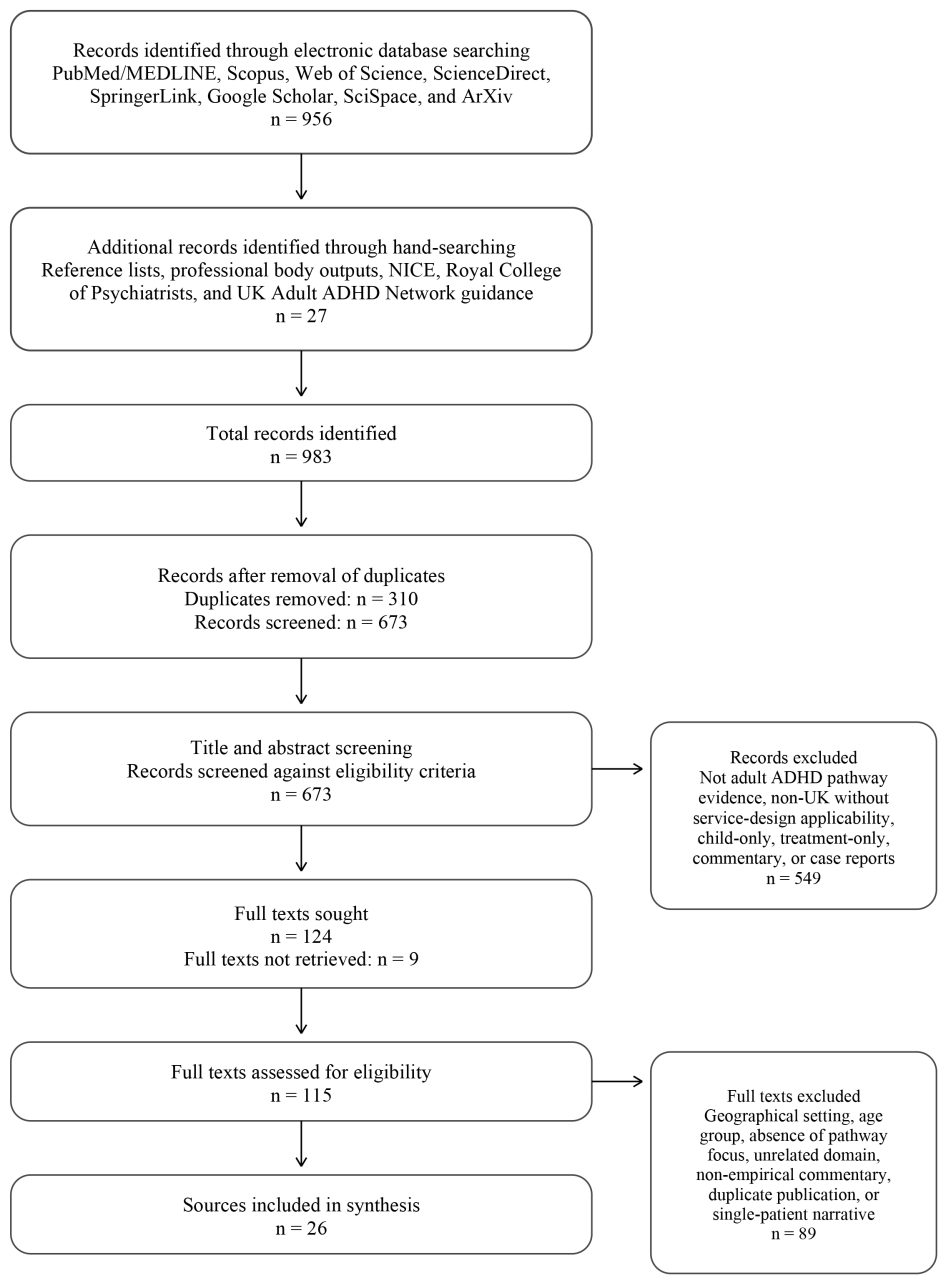
PRISMA-informed flow diagram of study identification, screening, and inclusion. The diagram is presented as a structured review flow diagram rather than as a formal PRISMA systematic-review diagram, reflecting the review design described in the Method.

The structured search identified 956 records from the eight electronic databases and 27 further records from hand-searching of reference lists, professional body outputs, and NICE, Royal College of Psychiatrists, and UK Adult ADHD Network guidance, giving a total of 983 records. After removing 310 duplicates, 673 records were screened against the title and abstract. At this stage, 549 records were excluded as not relating to adult ADHD pathways, being set outside the United Kingdom and not applicable to UK service design, addressing children only without separable adult data, focusing solely on treatment without diagnostic pathway content, or being editorials, commentaries, or case reports. Full texts were sought for 124 reports, of which 9 could not be retrieved. A further 89 reports were excluded at full-text assessment for reasons of geographical setting, age group, absence of pathway focus, unrelated clinical domain, non-empirical commentary, duplicate publication, or single-patient narrative. Twenty-six sources met the eligibility criteria and were included in the synthesis (**Figure 1**).

### Characteristics of included evidence

The included corpus comprised ten peer-reviewed empirical or mixed-methods studies, six service assessments, clinical audits, or quality improvement reports, five consensus statements or expert position papers, one systematic or scoping review with direct UK applicability, and four policy or guidance documents. Settings spanned NHS foundation trusts in England, community mental health teams in Wales, Scottish neurodevelopmental diagnosing teams, and national-level expert and professional body outputs. Publication dates ranged from 2013 to 2026, with most outputs published from 2020 onwards, reflecting both the acceleration of service research activity and the post-pandemic intensification of pressure on adult ADHD provision.

**Table 1** sets out each included source individually, grouped by evidence category, with its setting and design, sample or source type, pathway focus, and principal contribution, together with the nature of its evidential contribution. 

**Table 1:** Characteristics and contribution of included sources (n = 26).

Source [ref]	Setting and design	Sample or source type	Pathway focus	Main contribution	Nature of evidence
Peer-reviewed empirical and mixed-methods studies (n = 10)					
Maciver et al., 2025 (18)	30 neurodevelopmental diagnosing teams, Scotland; retrospective case-note review	408 adult neurodevelopmental assessment cases	Waiting times; service performance against time standards	Median adult wait 252 days (IQR 106–611); only 47% met the proposed 252-day standard; adults with ADHD waited longer than autistic adults	National descriptive evidence (Scotland)
Price et al., 2020 (19)	UK-wide national mapping survey across three stakeholder groups	2,686 respondents identifying 294 services	National service configuration; service mapping	44 dedicated adult ADHD services; 27% offered the full range of NICE-recommended interventions; discordance between stakeholder accounts	National descriptive evidence (UK-wide)
Price et al., 2019 (11)	UK methodological mapping paper	Service-mapping methodology	Methods for mapping service provision	Demonstrates that service mapping requires a dedicated method rather than routine system data	Indirect or contextual (methodological) evidence
Price et al., 2019 (20)	UK qualitative transition study	92 transition interviews	CAMHS-to-adult transition; information transfer	Poor information transfer and weak joint working impede transition	Transition evidence
Matheson et al., 2013 (21)	England qualitative study	30 adults with ADHD	Patient experience of service access	Access to diagnosis described as an uphill struggle within fragmented services	Patient-experience evidence
Morgan 2024 (22)	UK qualitative study	52 women diagnosed in adulthood	Gendered barriers; post-diagnostic support	Barriers for women referred from primary care and limited post-diagnostic support	Patient-experience evidence
Wills and Chakraborty 2026 (23)	UK qualitative study	52 UK-based females with ASD and/or ADHD	Late diagnosis; gendered barriers	Prolonged diagnostic processes, gender bias, and misdiagnosis	Patient-experience evidence
French and Cassidy 2026 (24)	UK qualitative study	20 late-diagnosed adults and clinicians	Experience of late diagnosis	Difficulties associated with late diagnosis of autism and/or ADHD	Patient-experience evidence
Jousselin 2018 (25)	Ethnography in an NHS adult ADHD clinic	Clinic ethnography	Waiting-list management	Rigid waiting-list management may disadvantage patients with ADHD-related impairments such as forgetfulness	Indirect or contextual evidence
William et al., 2024 (26)	UK mixed-methods study	46 survey respondents; 10 interviews	Post-diagnostic CBT provision	Difficulties with generic CBT provision after diagnosis	Patient-experience/local quality-improvement evidence
Service assessments, clinical audits, and quality improvement reports (n = 6)					
Agabani et al., 2022 (27)	North-east England CMHT service survey	Staff survey data	Waiting times; capacity; unmet need	Waits of 12–30 months for assessment and 6–36 months from referral to treatment initiation	Regional service evidence
Nyein and Oyewole 2021 (28)	Specialist adult ADHD clinic, Central and North West London; service assessment	115 patients offered specialist assessment	Waiting times; throughput	Shorter waits of 6–9 months in a dedicated specialist service	Local service/quality-improvement evidence
Streeter et al., 2023 (29).	GP referral pathway quality-improvement project	54 referral letters; 11 GP survey responses	Referral quality; triage	Childhood symptoms and functional impairment often incompletely described despite a high acceptance rate	Local audit or quality-improvement evidence
Gajdhar et al., 2023 (30)	East of North Wales CMHT clinical audit	50 confirmed adult ADHD cases	Assessment practice; standardised measures	Low documentation of standardised measures; variable use of multi-session assessment	Local audit or quality-improvement evidence
Corley and Hobson 2024 (31)	Paediatric ADHD service quality-improvement project (adult pathway relevance)	50 paediatric referrals	Triage tools (ASEBA questionnaires)	Illustrates the role of structured questionnaires in triage	Indirect or contextual evidence
Raja et al., 2021 (32)	North Wales adult ADHD clinics; quality assessment project	180 patients across six CMHTs	NICE compliance; physical health monitoring	Good diagnostic documentation but deficiencies in pre-treatment physical examination and side-effect monitoring	Local audit or quality-improvement evidence
Consensus statements and expert position papers (n = 5)					
Young et al., 2021 (12)	UK multidisciplinary expert consensus statement	Expert panel output	System-level barriers; national service failure	ADHD under-identified, under-diagnosed, and under-treated, with cultural and structural barriers	Professional consensus
Asherson et al., 2022 (33)	UK best-practice recommendations on mainstreaming into primary care	Expert panel output	Primary–secondary care interface	Three national constraints identified; case for primary care integration	Professional consensus
Smith et al., 2024 (9)	National service analysis/expert position	Expert authors	National service capacity	UK adult ADHD services described as being in crisis, with demand outstripping capacity	Professional consensus
Adamou et al., 2024 (13)	UKAAN quality standard development (AQAS)	Expert panel output	Assessment quality thresholds; assessor competencies	Defines minimum assessment quality standards to support clinical confidence and commissioning	Professional consensus with clinical-standard relevance
Leaver et al., 2023 (34)	Primary-care interface commentary	Expert authors	Shared-care prescribing; assessment quality	Concerns about variable assessment quality reaching primary care for shared-care prescribing	Professional consensus with contextual evidence
Systematic or scoping review with UK applicability (n = 1)					
Scarpellini and Bonati 2023 (35)	International scoping review with substantial UK relevance	23 qualitative studies	Transition; information transfer; joint working	Information deficits a recurrent barrier to transition; joint working and shared protocols proposed as solutions	Transition evidence
*Policy and guideline documents with direct pathway relevance (n = 4)*					
NICE NG87–2019 (15)	Statutory clinical guideline (England and Wales)	Guideline document	Diagnostic assessment; treatment; review	Normative benchmark for diagnosis and pathway quality	Clinical guideline or standard
NICE QS39–2018 (14)	National quality standard	Quality standard document	Quality indicators	Quality benchmarks for service delivery	Clinical guideline or standard
RCPsych CR235–2023 (16)	UK good practice guideline	Professional guidance document	Assessment; service organisation; prescribing	Good-practice benchmarks including comprehensive assessment, functional impairment, and collateral information	Clinical guideline or standard
Bolea-Alamañac et al., 2014 (36)	BAP evidence-based consensus guideline	Guideline document	Pharmacological management; prescribing	Prescribing and treatment benchmarks	Clinical guideline or standard

**Figure 2:**
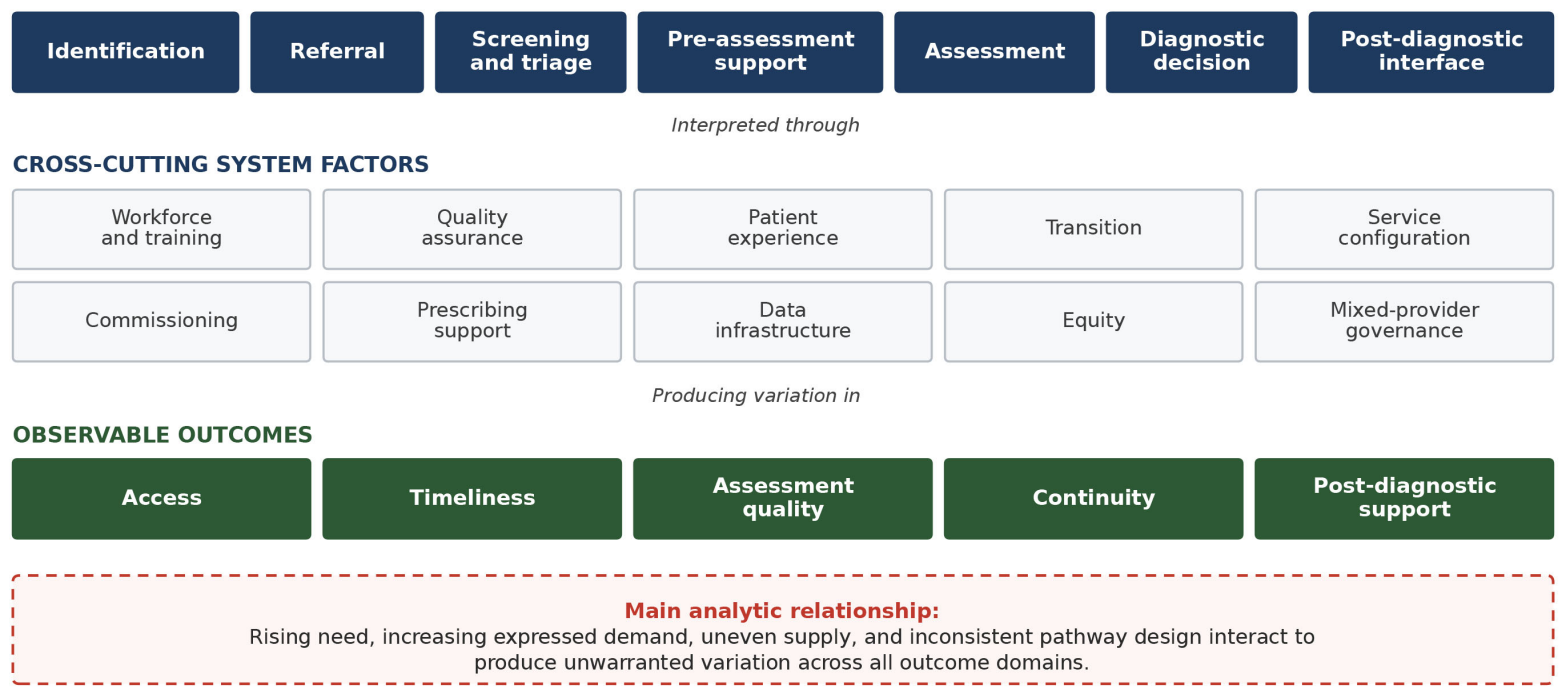
Conceptual organisation of the evidence synthesis. The figure shows how pathway stages, cross-cutting system factors, and evidence categories were interpreted together to support the commissioning and quality-framework argument.

The synthesis was organised around the stages of the adult ADHD pathway. These are identification, referral, screening, triage, pre-assessment support, assessment, diagnostic decision, and post-diagnostic interface. These pathway stages were interpreted through cross-cutting system factors: workforce and training; quality assurance; patient experience; transition; service configuration; commissioning; prescribing support; data infrastructure; and equity. The central analytic relationship was that rising need, increasing expressed demand, uneven supply, and inconsistent pathway design interact to produce variation in access, timeliness, assessment quality, continuity, and post-diagnostic support (**Figure 2**).

### Adult ADHD pathways as a mature service-system problem

National mapping and consensus work described adult ADHD provision in England as fragmented, geographically uneven, and constrained by limited specialist capacity (9, 12). Price and colleagues (19) identified 294 services across the UK, of which only 44 were dedicated adult ADHD services, and only 27% provided the full range of NICE-recommended interventions. Young et al. (12) concluded that ADHD is under-identified, under-diagnosed, and under-treated, with cultural and structural barriers operating across the healthcare system. Smith et al. (9) described UK adult ADHD services as being in crisis, with demand outstripping capacity and waiting times at unprecedented lengths. Asherson et al. (33) identified three interrelated national constraints: inconsistent interpretation of what constitutes a specialist, restriction of service delivery to limited-capacity secondary or tertiary care, and financial arrangements that impede the transfer of care between sectors.

Evidence on waiting times showed both prolonged delay and significant local variation. Maciver et al. (18) reported a median adult wait of 252 days across 30 Scottish neurodevelopmental diagnosing teams, with only 47 per cent of adult assessments meeting the proposed 252-day target and adults with ADHD waiting significantly longer than autistic adults. In this study, the proposed target and the observed median adult waiting time, although both equal to 252 days, are distinct quantities. The target represents the proposed maximum total-process standard, set *a priori* at 36 weeks, whereas the median is the empirically observed full waiting time for adults (interquartile range 106 to 611 days). The numerical coincidence is incidental and its practical implication is that the observed median sat at the proposed threshold, so that approximately half of adults waited longer than the standard. Consistent with this, only 47 per cent of adult assessments met the 252-day target. The benchmark, even where met at the median, therefore did not protect a substantial proportion of patients from prolonged delay.

Agabani et al. (27) documented waiting times for adult ADHD assessment ranging from 12 to 30 months and delays between referral and treatment initiation of 6 to 36 months in a north-east England CMHT survey. Nyein and Oyewole (28) reported shorter waits of 6 to 9 months in a specialist adult ADHD clinic in Central and North West London. Jousselin (25) described how rigid adherence to waiting-list management could disadvantage patients with difficulties, including forgetfulness.

### Unwarranted variation across pathway stages

Variation in access was evident in referral thresholds, accepted referrers, pre-referral requirements, and triage mechanisms. In some settings, adult ADHD remained embedded within general adult mental health structures. In others, more specialised or integrated pathways were available.

Variation in pathway content was evident in audit findings. Gajdhar et al. (30) found low documentation rates for standardised measures and variable use of multi-session assessment. Raja (32) and colleagues found good diagnostic documentation but deficiencies in pre-treatment physical examination and side-effect monitoring.

Variation in referral quality and triage was evident in Streeter et al. ‘s (29) audit of 54 referral letters, in which childhood symptoms and functional impairment were often incompletely described, despite a high referral acceptance rate.

Variation in continuity and ownership was evident at the interface between local NHS services, primary care, and non-local or independent-sector routes. Leaver et al. (34) identified concerns about variable assessment quality reaching primary care for shared-care prescribing.

Variation in transition was evident in the Scarpellini (35) review and in Price’s (20) qualitative transition study, both of which identified poor information transfer and weak joint working as recurrent problems.

### Clinical standards and operational delivery

Clinical standards for adult ADHD diagnosis were comparatively consistent across NICE guideline NG87 (15), NICE quality standard QS39 (14), Royal College of Psychiatrists CR235 (16), AQAS (13), and the British Association for Psychopharmacology consensus guideline (36). Across these sources, expectations were similar regarding comprehensive assessment, developmental history, functional impairment, corroborative information when available, and consideration of differential and co-occurring conditions.

By contrast, operational delivery was variable. Audit and service-assessment evidence showed inconsistency in the use of structured measures, corroborative history, physical health review, and follow-up processes (30, 32).

### Patient experience, waiting, and post-diagnostic support

Qualitative studies described access to adult ADHD diagnosis as difficult and protracted. Matheson (21) and colleagues described service access as an uphill struggle. Morgan (22) reported barriers for women referred from primary care and limited post-diagnostic support. Wills and Chakraborty et al. (23) described prolonged diagnostic processes, gender bias, and misdiagnosis. William et al. (26) identified difficulties with the provision of generic CBT after diagnosis.

### Infrastructure and visibility

The literature also described a weak infrastructure for understanding pathway performance. Price (19) found discordance between patient, clinician, and commissioner accounts of service availability, while their methodological paper showed that service mapping required a dedicated approach rather than routine system data.

## Discussion

The reviewed evidence suggests that adult ADHD pathways in England have reached a point at which local service redesign, while still necessary, is no longer sufficient as the primary policy response. The current landscape is marked by substantial unwarranted variation in access, timeliness, assessment quality, transition, and continuity. More importantly, these problems appear within a wider pattern of misalignment between underlying need, expressed demand, NHS capacity, and pathway design. When viewed together, these findings support the conclusion that England now requires a national commissioning and quality framework.

The distinct contribution of this paper is to move from description to architecture. Service mapping studies show that provision is uneven and fragmented (19, 37). Consensus statements show that the system is failing at scale (9, 12). Quality standards show what a defensible diagnostic assessment should contain (13–16, 36). However, none of those literatures, taken alone, specifies how an adult ADHD pathway should be commissioned as a whole. The present synthesis argues that, once these strands are read together, the appropriate policy response is not simply more capacity or better local practice, but a commissioning architecture capable of defining minimum pathway functions, minimum assessment standards, stratified allocation of expertise, and shared expectations across providers.

The case for a framework does not depend on claiming that every aspect of adult ADHD service delivery is already settled, nor on demonstrating that one exact pathway model is superior in every context. Instead, it rests on a more limited and more defensible proposition: that the system now has sufficient clinical consensus to define a minimum quality floor, sufficient service logic to support stratified care, and sufficient evidence of unwarranted variation to justify a national framework focused on functions, standards, and infrastructure rather than rigid organisational uniformity (13, 18, 19, 33).

Stratified care, as proposed here, is a principle for allocating expertise, not a triage mechanism for rationing access. No patient who meets the threshold for assessment is to be refused assessment under a stratified model. The question stratified care addresses is which level of expertise should be brought to which case, and at what point in the pathway. At the standard level, patients of lower clinical complexity, those without significant co-occurring mental illness, intellectual disability, substance use, forensic involvement, or diagnostic uncertainty, may be assessed within a less specialised pathway with specialist oversight available where required. At the specialist level, patients of higher complexity, with co-occurring conditions or diagnostic ambiguity, who may require multidisciplinary input can be assessed within a fuller specialist pathway. The criteria by which complexity is defined and redefined at the boundary of these strata are a matter for operational implementation; the framework requires that such criteria be made explicit and auditable, not that they be uniform across localities. A complementary framework for the stratification of adult mental health presentations more broadly is in preparation.

### Implications for commissioning

Adult ADHD pathways should no longer be treated as discretionary local service developments or narrow specialist-clinic functions. The reviewed evidence suggests that adult ADHD assessment requires explicit commissioning architecture across the whole pathway, including identification, referral management, screening and triage, pre-assessment support, diagnostic assessment, post-diagnostic communication, treatment initiation, shared-care arrangements, review, and re-entry routes for complexity or diagnostic uncertainty (33, 34).

This is especially relevant to financial planning. Where costs are calculated only around assessment throughput, services may appear productive while creating downstream pressure in primary care, medicines management, or secondary care review. Commissioning should therefore account for the full cost of diagnostic and post-diagnostic activity, including prescribing, monitoring, medication titration, psychoeducation, reasonable adjustments, non-pharmacological support, and review arrangements for complex cases. Otherwise, additional assessment capacity may convert a waiting-list problem into a continuing-care problem.

Integrated care boards also require a clearer basis for specifying what they are buying. At present, pathway descriptions may refer broadly to “ADHD assessment” while masking substantial differences in triage, assessment depth, collateral-gathering, diagnostic formulation, reporting, feedback, and post-diagnostic support. A national framework would reduce that ambiguity by specifying core pathway functions and minimum quality expectations.

### Core components of a national framework

The framework set out below is conceptual rather than operational and only defines the minimum pathway functions, minimum diagnostic standards, and expected forms of governance within which local services should be commissioned and configured. It does not yet specify operational criteria for stratification, fixed complexity thresholds, audit mechanisms, or rules for local implementation. These are matters for the implementation phase, in which the framework’s principles would be translated into operational specifications by national bodies, integrated care boards, and providers, with corresponding pilot and assessment work to refine them. The distinction is made here because the credibility of the framework rests on what it specifies (functions, standards, expectations, and lines of governance) rather than on what it leaves to local implementation.

**Table 2** translates the review findings into a proposed framework, linking each component not only to a general rationale but also to specific findings from the Results.

**Table 2:** Proposed components of a national adult ADHD commissioning and quality framework.

Framework component	Rationale	Concrete findings from the Results	Implementation risk
Minimum diagnostic standards	Protects diagnostic quality and consistency across providers	Stable standards across NICE, CR235 (16), AQAS (13), and BAP guidance (36) contrasted with variable operational delivery in (30, 32)	Overly rigid interpretation or checklist-based assessment
Core pathway functions	Ensures identification, referral, screening and triage, pre-assessment support, assessment, feedback, handover, prescribing support, and review are all commissioned	Variation in access routes, referral thresholds, triage practice, transition failures, and weak post-diagnostic continuity described across (19, 29, 35)	Local services may define functions differently without accountability
Stratified care	Matches assessment intensity to complexity and protects specialist capacity	Prolonged waiting and constrained specialist capacity described by (12, 18, 27, 33, 38)	May become crude gatekeeping if poorly governed
Workforce and competency standards	Addresses uneven expertise and supports safe delegation	Staff-reported training needs in (27) and competency expectations defined in (13) and (16)	Professional titles may be substituted for genuine competence
Shared data and outcome measures	Makes variation, quality, and pathway performance visible	Service-visibility discordance in (19) and reliance on bespoke mapping methods in (11)	Data collection may become burdensome or process-focused
Whole-pathway commissioning	Avoids assessment-only pathways and downstream prescribing or review bottlenecks	Post-diagnostic support gaps in (23, 26, 39); shared-care and transfer issues (28, 34).	Additional assessment capacity may displace pressure into prescribing or review
Equity safeguards	Ensures pathway redesign does not create new access barriers	Gendered and late-diagnosis barriers in (21–24) together with harmful or unhelpful generic post-diagnostic support in (26)	Efficiency reforms may favour articulate or administratively capable patients
Mixed-provider governance	Clarifies standards, reporting, handover, prescribing, and follow-up across NHS and independent routes	Shared-care concerns and assessment-quality variability described by (34), together with wider fragmentation described by (9) and (33)	Weak interface governance may create diagnostic and prescribing disputes

A national adult ADHD commissioning and quality framework should therefore contain at least these elements. It should define minimum diagnostic standards, specify core pathway functions, embed stratified care, define workforce and competency expectations, require a core pathway dataset and shared outcome measures, strengthen commissioning and financial transparency, ensure equity remains visible in design and monitoring, and address mixed-provider governance.

### Implementation risks and safeguards

A national framework would not remove implementation risk by any means. One risk is over-standardisation, in which national guidance is interpreted as a fixed service template rather than a specification of functions, standards, and outcomes. A second is that stratified care could become crude gatekeeping rather than proportionate allocation of expertise. A third is that needs-led support may become a substitute for clinically warranted diagnostic assessment. A fourth is quality dilution under pressure to reduce waits. A fifth is pressure displacement, in which increased diagnostic throughput shifts bottlenecks to treatment initiation, shared care, review, or re-entry. These risks do not weaken the case for a framework but indicate the conditions under which a framework must be designed and governed.

### Equity and unintended consequences

Equity should be treated as a design requirement rather than a retrospective performance measure. Several groups are at particular risk of disadvantage within current pathways. These are women and girls with internalised presentations, people with co-occurring autism or mental health conditions, people with substance misuse or justice-system contact, individuals with low health literacy, people without reliable informants, and those unable to complete detailed pre-assessment forms.

A national framework should therefore require equity impact assessment at each pathway stage. Referral criteria should be tested for whether they disadvantage people with poor documentation or limited GP advocacy. Triage processes should consider masked, internalised, or less visible presentations. Assessment standards should recognise that collateral history may be unavailable in adulthood and should specify acceptable alternative evidence. Post-diagnostic arrangements should not assume that patients can navigate fragmented systems unaided.

Equity also matters in relation to non-diagnosis. A high-quality pathway must be able to say both “yes” and “no” safely, and to explain either outcome clearly. If a person does not meet ADHD criteria, the pathway should still identify unmet needs, alternative explanations, and appropriate next steps.

### Future research and assessment agenda

The evidence base is sufficient to justify a framework response, but not sufficient to determine the best pathway model in all contexts. Future research should therefore move from describing service variation to comparing pathway configurations. Priority areas include comparative assessment of specialist, integrated neurodevelopmental, primary-care-supported, and mixed-provider models; studies of triage validity and safety; assessment of digital pre-assessment tools; economic analyses of full-pathway costs; and longitudinal outcomes following diagnosis, non-diagnosis, and delayed assessment.

Future work should also examine whether stratified care improves both access and quality, or whether it risks creating new inequities. This will require routine data that capture not only waiting times and diagnostic rates, but also patient experience, functional outcomes, treatment access, shared-care success, re-referral rates, and second-opinion rates.

There is also a need for better evidence and stronger governance at the interface between NHS and independent-sector pathways. The relevant question is whether all providers, regardless of sector, meet agreed standards for assessment quality, report content, prescribing governance, handover, and follow-up. In a mixed-provider landscape, quality assurance must follow the patient pathway rather than the organisational boundary.

The proposed framework is configuration-agnostic and is compatible with several alternative service models, including specialist adult ADHD clinics, integrated adult neurodevelopmental services, primary-care-supported pathways with specialist oversight, shared-care models, multidisciplinary assessment teams, digital pre-assessment and triage configurations, mixed NHS and independent-sector pathways, and regional networks operating tiered levels of expertise. The function of the framework is not to prescribe one of these configurations, but to define what each step must be commissioned to deliver and the quality standard to which it must be delivered. Local systems may continue to organise services differently, provided that the minimum pathway functions, assessment standards, stratified allocation of expertise, post-diagnostic continuity, and equity safeguards are observed. The framework is therefore best understood as a minimum national framework capable of supporting several legitimate local configurations.

### Strengths and limitations

We believe this paper has several strengths. It addresses an issue directly relevant to contemporary NHS service design. It integrates heterogeneous but complementary forms of evidence, including peer-reviewed studies, clinical audits, service assessments, qualitative work, professional guidance, service mapping studies, and national-level expert analysis. Screening and reporting were informed by PRISMA 2020 guidance, adapted for a structured literature review, and the selection process is transparent and reproducible. The review also moves beyond descriptive synthesis to make a more explicit commissioning and policy argument.

The limitations of the review should also be recognised. To begin with, the corpus of 26 included sources is modest and reflects the relative immaturity of the UK-specific adult ADHD pathway literature. Some sources, particularly conference-level service assessments and single-site audits, are methodologically weaker than full peer-reviewed studies and should be interpreted accordingly. Comparative implementation studies are limited, and the literature does not yet permit definitive claims that one exact pathway model is superior in every context. The review was not registered in advance, and quality appraisal was informal and consistent with a structured narrative approach rather than a formal systematic review.

It should also be stated that the restriction of the corpus to UK evidence limits the international generalisability of the proposed framework. The synthesis was constructed to inform a national policy argument for England, not to produce or to test a model that has been validated across health systems. The International relevance and transferability section identifies elements of the framework that appear conceptually transferable and those tied to features of the English NHS. However, these observations are conceptual rather than empirical. The framework should not be read as having been demonstrated to be effective in other jurisdictions, nor as having been evaluated against international service models. Readers in other health systems are therefore invited to consider the structural questions the framework raises, rather than to adopt its components directly.

### International relevance and transferability

Although this paper addresses the case for a national commissioning and quality framework in England, several of the pressures driving the argument are not unique to the English NHS. Rising adult ADHD referrals, prolonged waiting times, weak transition from child to adult services, uncertainty about the role of primary care, prescribing-governance difficulties, and variable post-diagnostic support are described in many health systems. The components of the proposed framework, therefore, admit two readings. Some elements are directly tied to the English context, including commissioning through Integrated Care Boards, the Right to Choose mechanism, NHS shared-care prescribing arrangements, and the governance of NHS-funded independent-sector provision. These elements are unlikely to transfer without translation.

Other elements are written at a level of abstraction that travels more readily. The notion of minimum diagnostic standards, the specification of core pathway functions from identification to post-diagnostic continuity, the stratified allocation of expertise according to clinical complexity, the requirement for whole-pathway commissioning rather than assessment-only investment, the use of a core pathway dataset and shared outcome measures, and the maintenance of equity safeguards across providers are all positions that other systems may consider. In jurisdictions that do not commission through a structure equivalent to an Integrated Care Board, the corresponding lever is national or regional quality governance. In jurisdictions that do not operate shared-care prescribing in its English form, the corresponding question is how primary and specialist care interfaces are aligned. In insurance-based systems, the corresponding question is how a payer specifies a recognised adult ADHD pathway and how it audits the quality and continuity of services across providers. The paper does not propose importing the English framework into other systems. It does propose that the structural questions the English case has surfaced of what an adult ADHD pathway is expected to deliver, by whom, to what standard, and under what governance are likely to be relevant in any health system facing comparable demand and capacity pressures.

## Conclusion

Adult ADHD diagnostic pathways in England have reached a stage at which the main challenge is no longer simply under-capacity, but the interaction between rising demand and a fragmented, variably configured, and only partially clear pathway landscape. The evidence indicates substantial unwarranted variation in access, timeliness, assessment quality, transition, prescribing support, post-diagnostic provision, and continuity of care. More importantly, it suggests that a deeper misalignment between need, demand, supply, commissioning, and infrastructure sustains these problems.

On this basis, England requires a national commissioning and quality framework for adult ADHD. Such a framework should be built around minimum diagnostic standards, stratified care, whole-pathway commissioning from identification through post-diagnostic support and treatment, active management of unwarranted variation, mixed-provider governance, and the infrastructure needed to make improvement visible and sustainable. It should not impose a single national service template. However, it should establish a minimally viable standard for what adult ADHD pathways are expected to deliver, while allowing local adaptation in how those functions are organised.

We want to add two further qualifications from our analysis. First, the evidence assembled here justifies the architecture of a national framework, but it does not yet identify the optimal service configuration within that framework. The question of which service model, for example, specialist clinic, integrated neurodevelopmental service, primary-care-supported pathway, mixed-provider arrangement, or regional tiered network, delivers the best outcomes within a national framework remains open and will require comparative implementation research. Second, the framework proposed here is conceptual rather than operational as it defines minimum functions, standards, and governance expectations but does not, at this stage, provide operational criteria for stratification, fixed complexity thresholds, audit mechanisms, or rules for local implementation. With these qualifications in mind, this paper argues that the policy starting point for adult ADHD in England is now the establishment of a national commissioning and quality framework and sets out the components such a framework should contain.

## Reflexivity

One of the authors has contributed to UK adult ADHD professional outputs cited in this review, including the Adult ADHD Assessment Quality Assurance Standard (AQAS). The cited standards and consensus statements are public documents authored by larger expert panels. They are used in this paper alongside independent national mapping evidence, peer-reviewed empirical studies, local audits, qualitative research, and policy documents. The categories of evidence used in the synthesis are described in the Method, and the inferential step from the synthesis to the policy argument is made explicit in the Discussion. Source selection followed the eligibility criteria stated in the Method rather than the authors’ professional networks. The authors have no commercial or financial interest in any of the cited standards or in the implementation of any framework derived from them.
